# Automated fluorescence intensity and gradient analysis enables detection of rare fluorescent mutant cells deep within the tissue of RaDR mice

**DOI:** 10.1038/s41598-018-30557-9

**Published:** 2018-08-14

**Authors:** Dushan N. Wadduwage, Jennifer Kay, Vijay Raj Singh, Orsolya Kiraly, Michelle R. Sukup-Jackson, Jagath Rajapakse, Bevin P. Engelward, Peter T. C. So

**Affiliations:** 1 Department of Biological Engineering, Massachusetts Institute of Technology, Cambridge, MA 02139 USA; 2 BioSystems and Micromechanics (BioSyM), Singapore MIT Alliance for Research and Technology (SMART) Centre, 1 CREATE Way, #04-13/14 Enterprise Wing, Singapore, 138602 Singapore; 3 Center for BioImaging Sciences, Department of Biological Sciences, National University of Singapore, Science Drive 4, Singapore, 117543 Singapore; 4 Department of Mechanical Engineering, Massachusetts Institute of Technology, Cambridge, MA 02139 USA; 5 School of Computer Science and Engineering, Nanyang Technological University, Singapore, Singapore

## Abstract

Homologous recombination (HR) events are key drivers of cancer-promoting mutations, and the ability to visualize these events *in situ* provides important information regarding mutant cell type, location, and clonal expansion. We have previously created the *Rosa26* Direct Repeat (RaDR) mouse model wherein HR at an integrated substrate gives rise to a fluorescent cell. To fully leverage this *in situ* approach, we need better ways to quantify rare fluorescent cells deep within tissues. Here, we present a robust, automated event quantification algorithm that uses image intensity and gradient features to detect fluorescent cells in deep tissue specimens. To analyze the performance of our algorithm, we simulate fluorescence behavior in tissue using Monte Carlo methods. Importantly, this approach reduces the potential for bias in manual counting and enables quantification of samples with highly dense HR events. Using this approach, we measured the relative frequency of HR within a chromosome and between chromosomes and found that HR within a chromosome is more frequent, which is consistent with the close proximity of sister chromatids. Our approach is both objective and highly rapid, providing a powerful tool, not only to researchers interested in HR, but also to many other researchers who are similarly using fluorescence as a marker for understanding mammalian biology in tissues.

## Introduction

Fluorescence imaging is now ubiquitous in the life sciences. While there are excellent approaches for studying sub-cellular processes using fluorescence, methods to study fluorescence in the context of thick tissue are lacking. For deep tissue analysis (more than $50\,\mu m$), the main challenge is the scattering of emission light that distorts image features. Although there are more sophisticated imaging techniques, such as multi-photon microscopy, these methods can be data intensive and prohibitively slow. Consequently, wide-field single photon microscope is the most commonly used imaging modality. Here, for wide-field microscopy, we have developed an algorithm that uses image intensity and gradient features to identify scattered foci deep within tissue. We have applied this approach to study rare fluorescent mutant cells in a mouse model designed to detect homologous recombination (HR) events.

Homologous recombination events are a key class of mutations^1^. While the HR pathway is generally accurate, HR-driven sequence rearrangements can cause deletions, insertions, translocations, and loss of heterozygosity (wherein information from one chromosome replaces that of its homolog). Virtually all tumors have undergone HR events that enable progression and metastasis. Understanding the molecular basis for DNA damage-induced sequence changes, is thus critical for understanding the underlying causes of cancer. Although much progress has been made using traditional tools of molecular biology and genetics in cell culture, few technologies have been developed for studying these important processes *in-situ*. To this end, we previously developed the *Rosa26* Direct Repeat (RaDR) mouse model that makes it possible to detect recombinant cells by a fluorescent signal^2,3^.

On the genetic level, to detect HR, RaDR mice harbor a transgenic fluorescence reporter. Specifically, these animals have a direct repeat of two truncated copies of the enhanced green fluorescent protein (EGFP) expression cassette. When sequence information is transferred from one cassette to the other, a full-length sequence can result, giving rise to a fluorescent signal. HR events are very rare (on the order of 1 in 10^5^ or 10^6^). To detect such rare fluorescent cells, flow cytometry has proven effective^4^. However, the frequency of fluorescent cells is not just a reflection of an HR event, but rather is the result of the combination of HR events and clonal expansion. To study HR, it is preferable to focus on HR events, and thus eliminate the effect of clonal expansion^4^. This can be achieved by imaging fluorescent cells within the context of a tissue, since HR events are rare (and thus spatially distinct) and cell division will result in the accumulation of fluorescent cells in the vicinity of the cell that had undergone HR, giving rise to a fluorescent focus. By quantifying the number of foci, instead of total cell number, the frequency of HR events and the impact of genes and exposures on HR can be more readily detected^4^. This approach has proved to be valuable for revealing how genes and environment affect the risk of sequence changes^3,5,6^.

While quantification of fluorescent foci within intact tissues has been shown to be an effective approach for studying HR^2,3,7,8^, this approach uses manual foci identification. It is slow and there can be significant variability among experimentalists. Moreover, manual counting of foci does not enable analysis of factors such as foci size, which is a valuable indicator of clonal expansion (providing insights into tissue physiology^9^). An automated foci-counting algorithm not only overcomes the variability of manual counting among researchers, but also greatly improves the efficiency of experiments. It may also provide means of estimating new quantitative measurements of the brightness and morphometry of each individual focus. Thus, automated algorithms potentially enhance our understanding of the factors that impinge on HR and on the extent of clonal expansion. From an image processing standpoint, foci counting is not a new problem and a number of image processing methods have been proposed over the last two decades. These image-processing algorithms can broadly be classified into foci quantification in organ/tissues or foci quantification in *in-vitro* cells. While automated foci counting in *in-vitro* cells is a well-established approach^10–12^, there are only a few studies on foci counting in tissue^13–15^.

**Figure 1:**
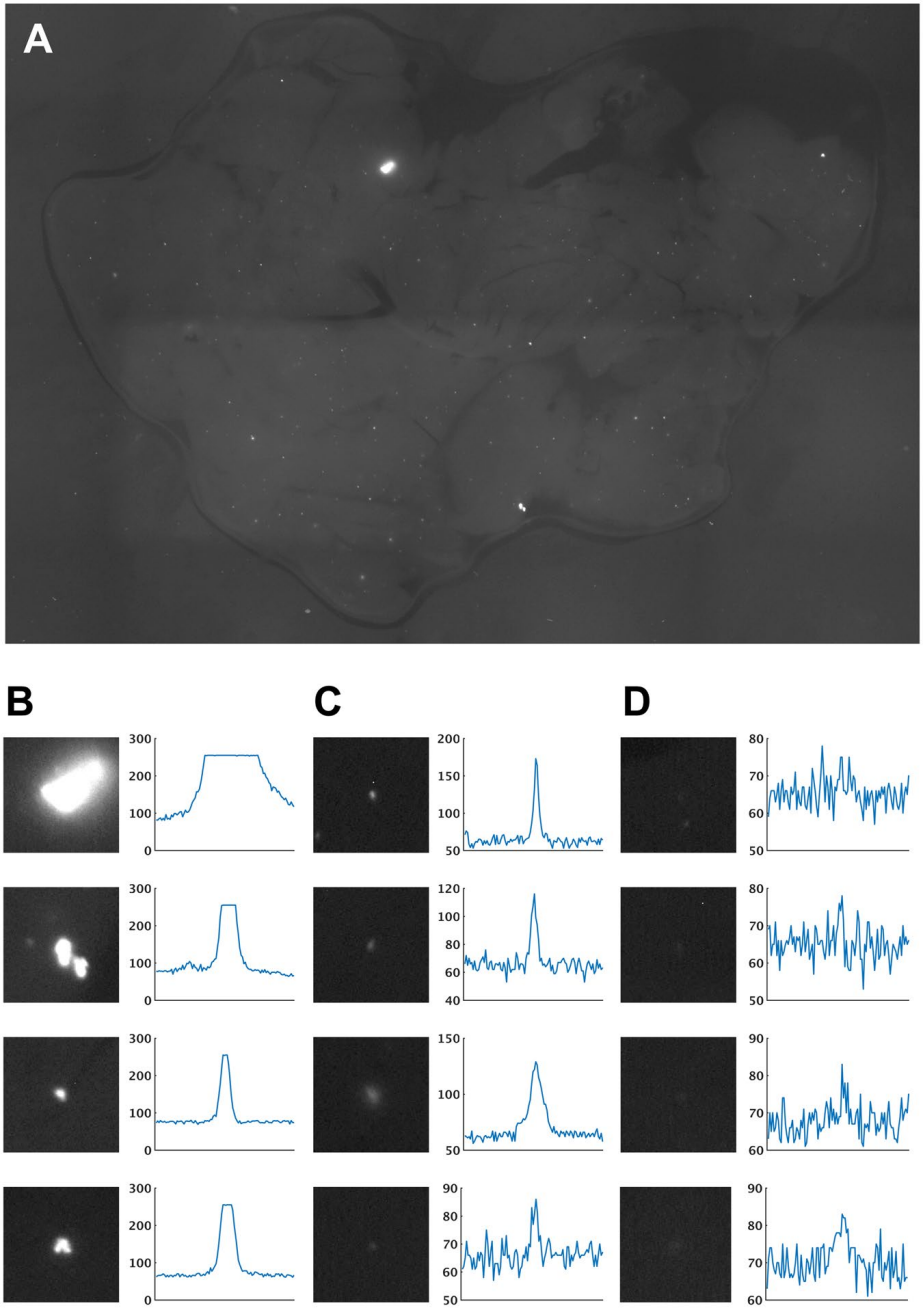
**(A)** A representative HR image from RaDR mouse pancreas. Bright foci are the cell clusters that have undergone HR. **(B)** Foci near the surface saturate the image sensor (left: Focus, right: cross-section at the mid-pixel row). **(C)** Foci at some intermediate depths aren’t saturated, but bright. **(D)** Foci in deep tissue are barely visible and are almost in the noise margin.

At the tissue level, effective programs for automated quantification of rare fluorescent foci within thick tissue were unavailable. This type of analysis poses its own unique challenges, like complex backgrounds, illumination variations^15^, and tissue scattering. Image processing is particularly challenging when basic wide-field microscopy at low magnifications is used. A representative image is shown in Fig. 1A. The imaged tissue contained fluorescent foci at different depths. Foci closest to the surface are saturated in the image (Fig. 1B). Ones at medium depths are not saturated, but appear as distinct bright spots with significantly higher intensities from their local backgrounds (Fig. 1C). Ones at larger depths appear blurred and dim compared to the first two types (Fig. 1D). As seen in the left column of Fig. 1D, they are almost invisible to the non-trained eye (a trained biologist, however, can identify them). The right column of Fig. 1D shows their respective intensity cross-sections. It can be observed that these foci are almost at the background intensity level and appear to be masked by the image detector’s noise.

It is worth noting that these challenges are unique to wide-field microscopy. Point scanning imaging techniques such as two-photon point-scanning microscopy or single photon confocal microscopy do not suffer from the above limitations. Point-scanning techniques do not illuminate the background; so as long as all the fluorescent photons are detected, they can be allocated to the corresponding imaging point. In our review of the literature, we did find programs for tissue-based foci counting using point scanning techniques, such as those that accompany confocal microscopy^13,14^. However, these imaging approaches are not feasible when one needs to analyze whole organs from multiple mice. The surface area per experiment is approximately 25 square centimeters, which is unwieldy using approaches that are effective for surface areas 100 times less. The problem is both the amount of time required to collect the image and the excessive amount of data that would need to be processed. In comparison, despite inferior image quality, wide-field microscopy is neither slow nor data intensive. Wide-field fluorescence microscopy has been used for segmentation of fluorescent objects in thin tissue sections (~10 *μ*m). For instance, Grigoryan *et al*. proposed an automated quantification method to count fluorescent spots resulting from fluorescence *in-situ* hybridization (FISH) labeling in tissue samples^15^. However, tissue specimens were only ~10 *μ*m thick. While wide-field fluorescent imaging has the advantage of unparalleled speed and simplicity, to the best of our knowledge, there is virtually no image processing algorithm developed for foci counting in wide-field 2D fluorescent images for thick (~500 *μ*m) tissue specimen.

In this work, we present a method for deep-tissue foci detection in wide-field fluorescence images. Our approach is suitable for analyzing fluorescent objects within intact freshly excised tissue. Importantly, tissues are not fixed, embedded and sectioned, but rather directly analyzed by imaging with a standard wide-field fluorescent microscope. Being inherently thick (~500um), these *in-situ* specimens contain foci at both shallow and deep tissue locations. Therefore, our approach utilizes both intensity and gradient information in the images; to help identify foci near the surface, we use intensity information through existing algorithms; to help identify foci in deep tissue, we introduce a new image gradient quantifier called *Focus-flow*. The two approaches, when configured to identify foci with weak responses, give rise to false positives. To separate false positives from real foci, we use a support vector machine. Experimentally, first, we validate our method *in silico*; results suggest that using gradient information increases detection accuracy for deep foci. Second, we test our method on real images from thick pancreatic tissue from multiple mice; results suggest that our method outperforms existing foci detection algorithms and is comparable to trained human raters. Last, we demonstrate our method in a study of HR events from heterozygous vs. homozygous mice; results confirm that HR is more frequent between sister chromatids compared to that between homologous chromosomes.

## Methodology

**Figure 2:**
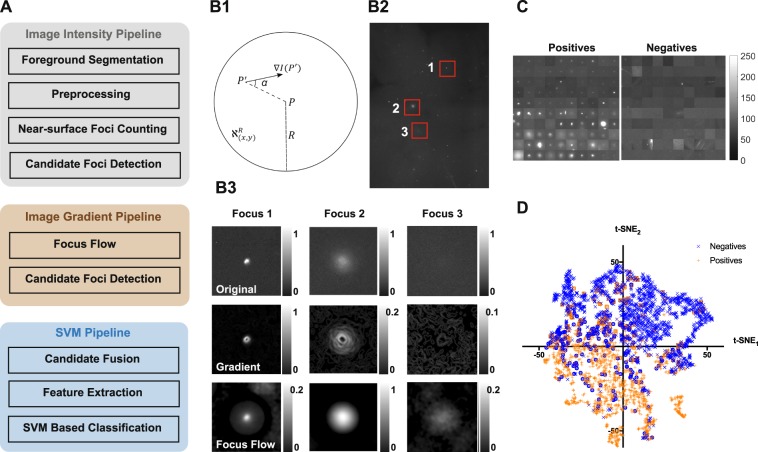
**(A)** The flow chart of the foci counting algorithm, consisting of the image intensity branch, the image gradient branch, and the SVM branch. **(B1)** A graphical representation of the filter used in the focus-flow in a neighborhood ${\aleph }_{(x,y)}^{R}$. $\nabla I(P\text{'})$ is the gradient vector at $P\text{'}=(x\text{'},y\text{'})$. **(B2)** Three foci at different depths on a tissue image. **(B3)** The three foci shown in ‘B2’ (top-row), their conventional gradients (middle row), and their Focus-flows. **(C)** A sample from the foci in the training dataset of 10 images. Each row contains a subset of training instances (positives and negatives) from each image. **(D)** The t-SNE plot of the training dataset applied to the SVM after training. (Incorrectly classified negatives are shown in blue circles and incorrectly classified positives are shown in orange squares).

The image analysis algorithm is summarized in the Fig. 2A. The algorithm is divided into three image processing pipelines; the image intensity pipeline, the image gradient pipeline, and the support vector machine (SVM) pipeline. Respectively, the image intensity and the image gradient pipelines are used to detect shallow foci and deep foci. But, this detection also misidentifies noise as foci. Therefore, in the SVM pipeline, real foci are separated from noise by employing a pre-trained SVM. The image intensity pipeline and the SVM pipeline mostly consist of existing image processing routines. Image gradient pipeline, however, contains a novel gradient quantifier termed *Focus-flow*.

In the next subsections, we first describe the *Focus-flow*. Then we discuss, in detail, the construction of the algorithm using the three pipelines. In the final subsection, we explain the SVM training procedure.

### *Focus flow* helps detect deep scattered foci

The major challenge of thick tissue imaging is tissue scattering. Tissue scattering distorts the emission light from fluorescent foci (see Fig. 1). The amount of distortion depends on the height of the scattering layer above the fluorescent focus. Therefore, compared to the foci in shallow tissue, foci in deeper tissue experience more scattering, and hence more distortion. The effect of this distortion is twofold; dispersion of the emission photons around the focus, and decay of the peak intensity of the focus. These effects respectively make deep foci appear blurred and dim. Blurring can be as high as hundreds of microns (over a few hundred-micron thick tissue layer); intensity decay can be as high as few orders of magnitudes (compared to that of a surface focus)^16^. Therefore, light from a fluorescent focus deep inside the tissue creates a very weak response on the camera. See Fig. 1B–D, that respectively show the intensity profiles of foci at the tissue surface, at some medium depth, and at a maximally detectable depth. At the surface, due to their high peak intensity, most of the foci saturate the image sensor (Fig. 1B). At an intermediate depth, although foci do not saturate the sensor, a clear intensity peak can be seen (Fig. 1C). At deep, due to decay, the foci are almost at the background level (Fig. 1D). Therefore, it is difficult to detect deep foci simply by looking at their intensity response. The information, however, is not completely lost. In fact, a trained biologist is capable of manually detecting these deep foci by carefully gauging their blurring effect. Similarly, here, we capture the information from deep foci by gauging their blurring effect through image gradient, which has proven to be useful to identify blob like objects in images^17,18^.

A deep focus, irrespective of how scattered, originates light from a specific locality. Therefore, in a given neighborhood, there should always be a gradient flow towards this locality. In other words, a pixel with a deep focus is expected to have the gradients of the pixels around it turned towards it. To capture this gradient flow, we designed a new quantifier called *Focus-flow*. Consider a pixel $P=(x,y)$ and it’s neighborhood ${\aleph }_{(x,y)}^{R}$ with a radius *R* (see Fig. 2B1). Let $\nabla I(x\text{'},y\text{'})$ be the gradient of an arbitrary pixel $P\text{'}=(x\text{'},y\text{'})$ in ${\aleph }_{(x,y)}^{R}$. Then the sum of the gradient towards *P* is given by,

1 $$g(x,y)={\sum }_{(x^{\prime} ,y^{\prime} )\in {\aleph }_{(x,y)}^{R}}\Vert \nabla I(x^{\prime} ,y^{\prime} )\Vert \times \,\cos ({\alpha }_{(x^{\prime} ,y^{\prime} )}).$$

Here, ${\alpha }_{(x^{\prime} ,y^{\prime} )}$ is the angle between, the line *PP*′ and the direction of gradient vector $\nabla I(x^{\prime} ,y^{\prime} )$ (see Fig. 2B1). Then we define the *Focus-flow* as the ratio between $g(x,y)$ and the total gradient in the neighborhood,

2 $$Focus\,Flow(x,y)=\frac{g(x,y)}{{\sum }_{(x{\rm{^{\prime} }},y{\rm{^{\prime} }})\in {{\rm{\aleph }}}_{(x,y)}^{R}}\parallel {\rm{\nabla }}I(x{\rm{^{\prime} }},y{\rm{^{\prime} }})\parallel }.$$

The only parameter of *Focus-flow* is the radius of the neighborhood, *R*. For our images we selected *R* as 30 pixels or equivalently 128 μm (at our magnification and camera pixel size). It is large enough to capture most of the blurred foci up to a few hundred micrometer depth (see supporting Fig. S8B); it is mostly small enough not to mix neighboring foci.

In order to see the impact of Focus Flow, consider the three representative foci shown in Fig. 2B2. They are shown separately in the first row of the Fig. 2B3. The pixel values in the figure are normalized to the maximum intensity of the corresponding image. The second row of the Fig. 2B3 shows the simple gradient of the three foci. While for focus 1 and focus 2 some distinct response above their background level can be seen, for focus 3 the response is almost at the background level. Please note that the scales, on the right of each plot, are different. It is also worth noting that the simple gradient follows the intensity response (foci1 > foci2 > foci3). In contrast, the *Focus-flow* does not follow intensity response (bottom row of Fig. 2B3). The highest *Focus-flow* response is seen for focus 2 and then respectively for focus 1 and focus 3. More importantly, the response of *Focus-flow* is significantly above the background for all three foci. However, since there is no gradient on the saturated area, the *Focus-flow* is not a suitable quantifier for saturated foci (see supporting Fig. S1). These foci should be detected using their intensity.

### Final foci detection algorithm combines intensity, gradient and morphological information

The final algorithm consists of three image processing pipelines; the image intensity pipeline, the image gradient pipeline, and the SVM pipeline. In the image intensity pipeline, we first segment the foreground tissue region (supporting Fig. S2A) using an active contour or “snakes” based algorithm^19–21^ (the algorithm was borrowed from^22^). If necessary, the user can also make corrections (supporting Fig. S2B) at a later stage using our graphical user interface (supporting Fig. S3). Then the image is preprocessed by applying an averaging filter followed by a median filter.

Foci near the surface are expected have considerably higher image intensity than their background. Since near-surface foci mostly comprise of ballistic photons, they also have clear foci boundaries and hence can be segmented. Here we use a local maxima extraction algorithm named extended maxima transform (EXMAX)^23^ to identify and segment near-surface foci. EXMAX is a single-parameter algorithm. The parameter, *h-value*, often dictates the resulting segmentation (see supporting Fig. S4A). The best *h-value* that gives rise to the highest counting accuracy (compared to the manual raters) seemed to vary between the images (see supporting Fig. S5). Therefore, we use a heuristic algorithm to adaptively set the *h-value* that results in a realistic foci segmentation for each image. More details about the heuristic algorithm can be found in the section S1 in the supporting text.

It is practically inefficient to tune the heuristic algorithm to cater to all the various images (see supporting Fig. S5). We use a different approach where we overestimate the number of foci (we call the overestimated results, *foci candidates*) and later separate the positives (real foci) from negatives (noise) using a classifier. The foci candidates are selected by applying the EXMAX transform with a low *h*-value. The *h*-value is selected as the lowest value that allows less than “*n*” times the number of foci as the heuristic count. The over-counting ratio, “*n*”, is an input parameter. We set *n* to 2 (higher values of *n* increased the number of candidates but did not improve the final counting accuracy).

In the image gradient pipeline, the *Focus-flow* is first calculated. Then the same candidate detection process explained above is applied on the *Focus-flow*. Again, the over-counting ratio was set to 2 for the same reasons above.

Image intensity and gradient pipelines, each results in a binary image with foci candidates. In the SVM pipeline, first the two images are fused together. Here the gradient candidates, that intersect with intensity candidates, are removed to avoid any merging. Then the two binary images are fused together by performing pixel-wise OR operation. Second, the intensity image, focus flow, and the fused candidate image are used to extract eighteen features for each candidate focus. The features are listed in the supporting Table S1. Each candidate focus is now represented as an eighteen-dimensional vector. These feature vectors are then fed to a pre-trained support vector machine (SVM) via principal component analysis (PCA). Data before and after PCA are shown in supporting Fig. S6. The SVM separates the foci candidates into positives (real foci) and negatives (the noise picked up by EXMAX during candidate detection). The SVM was trained using images annotated by a trained biologist. The training process is explained in the next section.

### SVM training

During the initial training process, twenty images, that together contained roughly 8000 foci candidates (~4000 real foci, i.e. positives and ~4000 negatives) were used. The images were divided into two sets as, ten training images, and ten testing images. Fig. 2C shows a random subset of the positives and negatives. Foci candidates shown in each row were randomly selected from each training image. We selected an SVM with a radial basis function (RBF) kernel. The two hyper-parameters, *C* and *γ* (see supporting section S2 and ref.^23^ for more details), were trained in a leave-one-out fashion on the ten training images. Briefly, a grid of *C* and *γ* values with exponential increments were created. Then for each combination of *C* and *γ* the following tainting process was performed. One image was separated from the training set and the SVM with the current *C* and *γ* was trained on the other nine training images. The training accuracy (Eq. 3) was recorded. The same process was repeated ten times leaving a different image out each time. Then the average accuracy was calculated over the ten iterations. This process was repeated for all the combinations of *C* and *γ* in the grid. The *C* and *γ* combination that resulted in the highest average accuracy was then selected. Last the SVM with the selected hyper-parameters (*C* and *γ*) was trained on all ten training images. Fig. 2D shows the t-sne^24^ plot of all the foci candidates in the training image set.

### Ethical approval

All animals were housed and handled in Association for Assessment and Accreditation of Laboratory Animal Care (AAALAC)-accredited facilities with diets, experimental methods, and housing as specifically approved by the Institutional Animal Care and Use Committee. The MIT CAC (IACUC) specifically approved the studies as well as the housing and handling of these animals. All methods were performed in accordance with the relevant guidelines and regulations.

## Results and Analysis

The algorithm was tested on both synthetic images and pancreas images from multiple mice. Then, the algorithm was demonstrated in an analysis of HR events in pancreas, liver, and colon from two types of mice.

### Results on synthetic images suggests improved accuracy in deep foci detection

**Figure 3:**
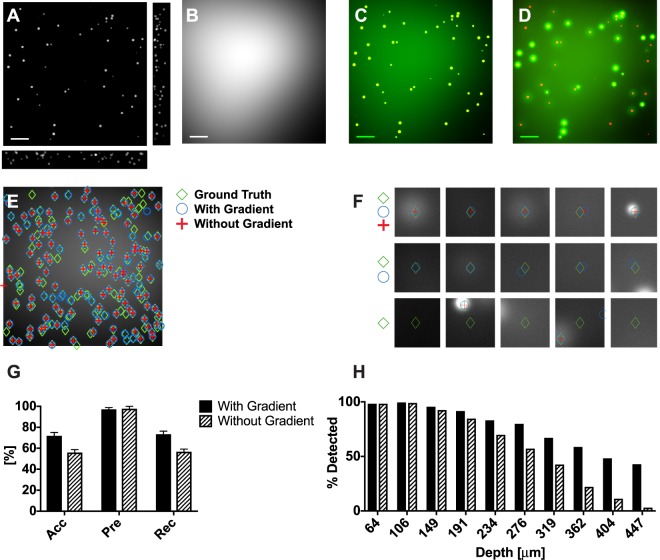
**(A)** Simulated thick tissue section with a random distribution of foci with a random number of cells in each focus. **(B)** Simulated heterogeneous auto-fluorescence background. **(C)** Simulated image of the tissue section in ‘A’ in the absence of scattering (green channel). Foci are shown in the red channel. Most of the foci are visible despite background. **(D)** Simulated image of the tissue section in ‘A’ in the presence of tissue scattering (green channel). Foci are shown in the red channel. Some foci are buried in the background (see the strong red). Scale bars are 1 mm. **(E)** Results of foci counting with (blue circles) and without (red crosses) gradient information. Shown in green diamonds are the ground truth foci locations. **(F)** Three subpopulations of foci. The top row shows foci detected with gradient information as well as without gradient information. The middle row shows foci detected with gradient information but weren’t detected without gradient information. Shown in the bottom row are foci that weren’t detected. **(G)** Accuracy, precision, and recall in foci detection with and without gradient information. **(H)** Percentage of foci detected with and without gradient information plotted against their depth. Including gradient information improved the foci detection at deeper locations.

We first tested the algorithm on a simulated data set to quantitatively gauge its aptness to detect deep tissue foci. A set of twenty fluorescent-foci images were simulated using the following method. For each image, a set of 200 fluorescent cell clusters were randomly distributed in a tissue object of 10 × 10 × 0.5 mm^3^ volume (Fig. 3A). Each cluster contained a random number of cells and each cell contained a random fluorophore concentration. Then, a non-uniform autofluorescent background was simulated over the volume of the object (Fig. 3B). In the absence of any scattering, the imaging process can be modeled by convolving the tissue object with the point spread function (PSF) of the imaging microscope. Therefore, we modeled a wide-field microscope’s PSF (see supporting section S3) and convolved it with the tissue object. A representative image synthesized is shown in the Fig. 3C. This image doesn’t contain any tissue scattering.

To model the light scattering process in tissue, a scattering point spread function (sPSF) was simulated at each depth using Monte Carlo methods (see supporting section S4). At each depth plane, the tissue volume was convolved with the respective sPSF to generate the fluorescent intensity profile at the tissue surface. Finally, the intensity profile at the tissue surface was convolved with the PSF of the simulated wide-field microscope. Fig. 3D shows the resulting simulated image in the presence of scattering (compare with the non-scattering case, i.e. Fig. 3C).

The twenty simulated images were then used on the algorithm. Ten images were used for training and ten were used for testing (similar to explained in the Methodology section). The only differences were, the images being synthetic, and the ground truth having derived from the simulation process. In order to test the contribution from the gradient pipeline, the same process was repeated without the gradient pipeline. Fig. 3E shows a representative image after processing with and without the gradient. Fig. 3F shows some representative foci from the same image. The foci in the first row were detected by both versions of the algorithm, i.e. with and without gradient. The foci in the second row were not detected by the version without the gradient pipeline. The foci in the last row were not detected by either version. We compare the average accuracy (Eq. 3), precision (Eq. 4), and recall (Eq. 5) of the two versions in the Fig. 3G.

3 $$Accuracy=\frac{TPs}{TPs+FNs+FPs}$$

4 $$Precision=\frac{TPs}{TPs+FPs}\,$$

5 $$Recall=\frac{TPs}{TPs+FNs}$$

Here TPs, FNs, and FPs denote the number of true positives (real foci and were detected by the algorithm), false negatives (real foci but weren’t detected by the algorithm), and false positives (not real foci but were detected by the algorithm). The precision of both versions were high and similar in value suggesting that there are fewer false positives in both versions. The accuracy and the recall of the version with the gradient pipeline were more than 15% higher compared to that without the gradient pipeline. This observation suggests that the inclusion of the gradient pipeline reduced FNs and intern, improved foci detectability. Finally, in Fig. 3H, we plot the percentage of detected foci at each depth over all the images. As expected the version of the algorithm with the gradient pipeline gradually improved the detection percentage with increasing depth.

### Results on tissue images demonstrates the algorithm’s agreement with human raters

We tested the proposed algorithm on real data from mouse pancreatic tissue images and compared the results with existing methods. First, we briefly explain the sample preparation process. Animals were euthanized with CO_2_ according to AVMA guidelines. Tissues were excised and held on ice in tubes containing PBS with 0.01% trypsin inhibitor (T9008. Sigma-Aldrich) until use. The entire pancreas was collected. Tissues were then compressed to 0.5 mm between coverslips and imaged for EGFP under the 1x objective with the FITC filter of a Nikon 80i fluorescent microscope.

Twenty pancreas images were acquired from tissue from twenty animals. The images were then analyzed by two trained biologists. One biologist (the training rater) was asked to detect foci and annotate the images. The other biologist (the independent rater) was asked to count the number of foci in the images according to the usual protocol and report the counts. The annotations of ten images by the training rater was used to train the algorithm according to the process explained in the Methodology section. The other ten images were used for testing. We also processed the ten testing images with an existing algorithm named, “Find foci”^25^ for benchmarking. Find-foci is a fully automated foci detection algorithm developed for microscopy images of biological specimens; its parameters can be trained with a set of manually annotated images, similar to our proposed algorithm^25^.

**Figure 4:**
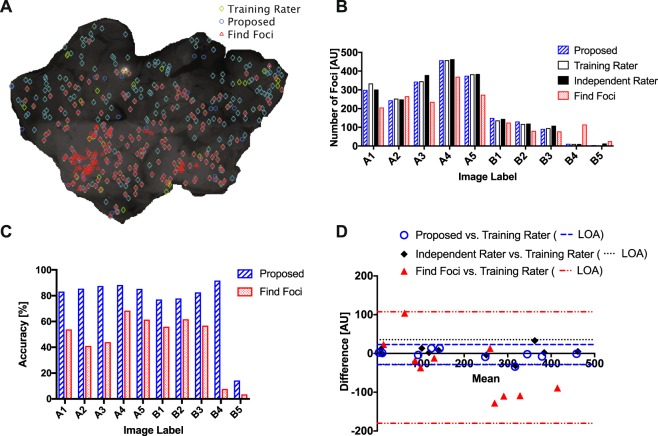
**(A)** Foci counting results for a representative pancreatic tissue image using: manual foci detection, with the proposed algorithm of this paper, and a published algorithm called *Find Foci*. **(B)** The foci counts for ten test pancreatic images counted by: the proposed algorithm, two manual raters (the training rater and an independent rater), and *Find-foci*. The agreement between the two manual raters and the proposed algorithm is considerably higher compared to that of with *Find-foci*. **(C)** The accuracy of the proposed algorithm and *Find-foci* for the same ten images as in ‘B’. Here the training rater’s foci locations were treated as the ground truth. The proposed algorithm’s average accuracy was ~77% while *Find Foci*’s was ~45%. **(D)** Bland-Altman plot of the difference vs. mean for counts: between the proposed algorithm vs. the training rater; between the independent rater vs. the training rater; and between *Find-foci* vs. the training rater. Shown by dotted lines are the respective 95% Limits of Agreements (LOA).

On a representative image, Fig. 4A shows the foci detected by the three methods: manual foci counting (by the training rater), the proposed algorithm, and Find-foci. Qualitatively speaking, the training rater and the proposed algorithm showed reasonable agreement, while Find-foci seemed to miss real foci and detect a number of false positives. The final number of foci counted by the proposed algorithm, the training rater, the independent rater, and Find foci are shown in the Fig. 4B. Again, while the former three demonstrated similar agreements, Find-foci showed a significant deviation from them. In order to quantitatively compare the performance of the proposed algorithm and Find-foci, we treated the annotations from the training rater as the ground truth and calculated the detection accuracy of the two algorithms for each image (Fig. 4C). The average accuracy of the proposed algorithm was 77%. In comparison, Find-foci’s average accuracy was only 45%.

Moreover, the proposed algorithm demonstrated closer to 80% accuracy for all images except for one image (B5 in the Fig. 4C). According to the training rater, the image contained only three real foci; the proposed algorithm detected five; the benchmarking algorithm Find-foci counted 27; the independent rater counted 14. Thus it’s evident that there’s no universal ground truth and the four raters, including the two human raters, do not agree most of the time (see Fig. 4B for other instances). In order to quantitatively analyze the agreement between the raters, in Fig. 4D we show the Bland-Altman plot of the difference vs. mean^26^ for the agreements: between the proposed algorithm and the training rater; between the independent rater and the training rater; and between Find-foci and the training rater. The respective 95% limits of agreements were 53 foci, 64 foci, and 288 foci. Thus it’s evident that the agreement between the proposed algorithm and the training rater was similar to the agreement between the two human raters. The agreement between Find-foci and the training rater, however, was about five fold worse than that of the proposed algorithm and the human raters.

### Differences in HR in tissue from heterozygous and homozygous mice

In this section, as a demonstration of the algorithm, we present the results of a study of homologues recombination (HR) event frequencies of heterozygous vs homozygous mice. The mice that were used in the previous study were heterozygous for the HR substrate. We predicted that mice containing copies of the HR substrate on both chromosomes would have approximately double the frequency of fluorescently detected recombination events observed in mice containing only one copy of the HR substrate. Specifically, the HR substrate contains two copies of the EGFP gene sequence, where each copy has been truncated at either the 5′ or 3′ end so that the protein products do not fluoresce. During DNA replication, the sister chromatids will be in proximity, each containing the HR substrate. If a DNA double-strand break occurs in one copy of the HR substrate, it may be repaired by HR using the sister chromatid as a template for repair. If HR occurs between the cassette harboring the 5′ truncation and the cassette on the sister chromatid that has the 3′ truncation, full-length protein sequence can be restored. Expression of the full-length sequence enables the production of a fluorescent product [Figs 1 and 3 of the ref.^27^], which is visible by fluorescence imaging, as described above.

**Figure 5:**
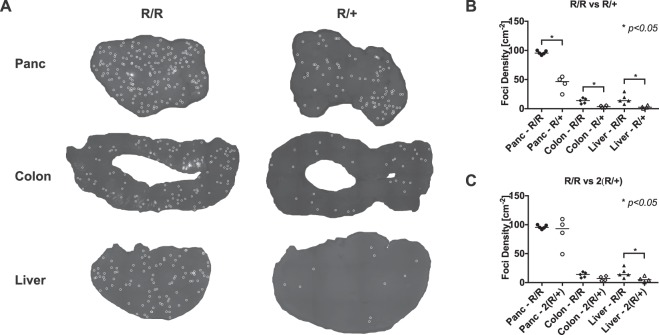
**(A)** Resulting foci annotation from the algorithm for representative samples of the pancreas, the colon, and the liver from homozygous (R/R) and heterozygous (R/+) mice. **(B)** Foci densities for each tissue type from R/+ and R/R mice. R/R shows a higher number of HR events for all tissue types (*$p < 0.05$). **(C)** Foci densities of R/R and 2-fold R/+ (denotes as 2(R/+)) for each tissue type. While for pancreas and colon R/R and 2(R/+) show similar foci densities, for liver R/R is higher.

Somatic chromosomes come in pairs, one homologous chromosome from each parent. In the case of the RaDR mice that are heterozygous (R/+), there is a copy of the HR substrate on one copy of Chromosome 6, while the other copy of Chromosome 6 remains wild type. For homozygous (R/R) mice, there are two copies of the HR substrate, one on each copy of Chromosome 6. Therefore, it is expected that the frequency of fluorescent foci would be at least twice as high in the R/R, as compared to heterozygous mice. To explore this possibility, tissue specimens from pancreas, liver, and colon were prepared and imaged. Tissue samples were prepared in the same way as explained in the previous section. The entire pancreas was collected, as well as the left lobe of the liver. The entire colon was excised (cecum to anus), cut open on one side, and the lumen was rinsed of fecal matter before placing in PBS + trypsin inhibitor. The algorithm was first trained and used for liver and pancreatic images, enumerating all fluorescent foci. For the colon, it is biologically important to distinguish between types of foci: the colon contains somatic stem cells that divide to give rise to the epithelium, and those cells are sloughed off over time (called “transit cells”). Transit cells in this mouse model produce small, irregularly shaped foci, whereas mutations in stem cells produce larger, brighter, and roughly circular foci. Therefore, the algorithm was trained to differentiate between large clusters of fluorescent cells that arise from a stem cell mutation (where nearly all cells are fluorescent due to clonal expansion), versus recombinant transit cells. For details, please see Sukup-Jackson *et al*.^27^. For all tissue types, the foci counts were recorded and the foci densities (foci per cm^2^) were calculated. Fig. 5A shows resulting foci detected in pancreas, liver, and colon for both R/+ and R/R animals. Mann-Whitney U-test was conducted between groups using GraphPad Prism 5 (the distribution of EGFP-positive cells in RaDR mice is non-normal across tissues and among individual animals; therefore, a nonparametric rank comparison test is appropriate). By eye, it is clear that the R/R mice have more foci in all three tissue types. Fig. 5B shows the foci densities of R/+ and R/R animals for each tissue type. As anticipated, the foci density for R/R animals was consistently higher than that for R/+ animals.

Published studies show that recombination between sister chromatids is more than 10 fold more frequent than recombination between homologous chromosomes^28^. This is in part because sister chromatids are positioned next to each other following DNA replication. If it is the case that relatively few recombination events occur between homologous chromosomes, then when we multiply the data for the heterozygous mice by 2-fold, we would expect that the frequency for R/R and 2(R/+) to be similar. As can be seen in Fig. 5C, for pancreas and colon, the median frequency values were very similar. From these, we can conclude that recombination between homologous chromosomes is indeed a minor contributor to recombination at the HR substrate. Interestingly, there is a statistically significant increase in the frequency of HR in the R/R as compared to 2(R/+) for the liver, which could indicate a different preference for HR between chromosomes compared to the other two tissues. However, the number of samples for the two cohorts is quite low; so this could just be a result of variability in the data. Nevertheless, it would be very interesting if the ratio of HR between sisters versus between homologous chromosomes is tissue or cell-type dependent.

## Discussion

When propagates through more than $\sim 50\,\mu m$ in tissue, light encounters scattering which distorts images in fluorescence microscopy. Optically, this issue is overcome through the use of two-photon point scanning imaging^16^. Two-photon microscopy, however, requires complicated instrumentation and is extremely slow compared to single-photon wide-field microscopy. Therefore, despite scattering, single-photon wide-field microscopy is the most practical imaging modality for large tissue volumes, with imaging regions in the order of tens of square centimeters. One such example is organ-wide HR event detection in animal models^2,3^. Each HR event gives rise to a fluorescent cell cluster that is seen as a focus in microscopy images. Foci closer to the tissue surface can be easily detected but foci at deep tissue locations generate weak blurred signals that could only be detected by trained biologists. Existing automated image analysis algorithms, having designed for non-scattering imaging conditions, do not perform well for these images (see the results on synthetic images in the Results and Analysis section). In this paper, we present an automated foci detection method specifically designed for wide-field fluorescent images of thick ($\sim 500\,\mu m$) tissue sections.

Using an automated algorithm presents a number of advantages over manual foci counting. Manual foci counting is subjective. Different experts may gauge the image features differently (see our results in Fig. 4B and D); an automated algorithm overcomes this limitation. Second, the manual foci counting process is laborious and time-consuming. An expert biologist may take days to weeks to carefully analyze these images. An automated algorithm not only helps reduce human workload but also speeds-up the analysis process; the algorithm currently takes only minutes to hours, based on the size of the dataset. We also stress that we haven’t done any computational optimization. One may parallelize most of the image processing routines, for an example using graphical processing units (GPUs), and scale down the processing time to minutes or even to seconds. Another advantage of computer-based analysis is its ability to enable analysis of subtler image features such as foci size, shape, and brightness. These image features may be linked to tissue features through mathematical modeling. However, we note that deep foci cannot be segmented in a conventional sense; tissue scattering loses high-frequency information in images and hence prohibits recognizing exact foci boundaries. But one may still make estimates through a scattering model.

To detect foci, our algorithm uses both image intensity and image gradient information. The image gradient is quantified using a new technique called Focus-flow. *In silico*, we demonstrated that Focus-flow improves the overall foci detection accuracy by more than 15% (see results on synthetic images in Results and Analysis). Furthermore, our results suggest that inclusion of Focus-flow improved detectability by 100–300% for deep foci (Fig. 3H). For *in-situ* pancreatic tissue images, we demonstrated that our method is comparable to trained human raters (see results on tissue images in Results and Analysis). Compared to a trained biologist our algorithm demonstrated closer to 77% average accuracy (Fig. 4C). In comparison, Find-foci, a benchmarking algorithm designed for automated foci detection, was only 45% accurate (Fig. 4C). Manual foci counting is subjective. Different human raters may generate different results. Therefore, we compared the results from another independent rater with the results from the first human rater. The two demonstrated a considerable variation; the limit of agreement (LOA) in Bland-Altman analysis was 53 foci. The algorithm and the first rater demonstrated similar agreement while the LOA between Find-foci and the first rater was almost fivefold worse (Fig. 4D).

Our algorithm uses a support vector machine to separate real foci from false positives. Therefore, it requires training instances from human raters. On one hand, the results will only be as good as the training data. An algorithm trained with a set of images at a limited number of conditions may work only for images taken at similar conditions. Therefore, the training process must be rigorously administered. On the other hand, this approach has a number of practical advantages. First, it helps to reduce the number of free parameters in the overall image processing pipeline. Our pipeline contains only two free parameters; with both fixed, the algorithm worked robustly over a large set of experiments. The intended users, therefore, require little to no image processing expertise; all users have to do is to annotate foci in a set of training images and feed them to the algorithm in the training mode. Second, the algorithm can combine training data from multiple experts, so that there is a balanced agreement for all raters and hence remove any subjectivity in the results. Last, the algorithm can be trained for different user requirements. For, an instance for colon it is biologically important to count only the mutations in stem cells and disregard transit cells (see last subsection in Results and Analysis). Though there are obvious differences between the two types, the ultimate decision necessarily is a fuzzy one. For a conventional parametric algorithm, one would need to carefully define the separation of the two cell types with respect to each parameter. This obviously hinders the computational plasticity of the algorithm for new conditions. But, in our approach, the experts had an easy comprehending approach to transfer their domain knowledge.

## Conclusion

In this paper we present an automated algorithm to quantify rare fluorescent foci in wide-field images of *in-situ* thick tissue. We tested our algorithm *in silico* as well as in real experiments. *In silico* experiments suggested that the new method improved deep foci detectability by a factor of two to three. We also validated our method via comparisons with multiple experts in HR image quantification. The results suggest that the proposed algorithm performs similarly to an expert human rater. The use of our new analysis program has significant advantages over manual counting, due not only to providing improved consistency and far greater speed, but also because it provides a foundation for analysis of subtler parameters that cannot be estimated by eye, such as foci size, shape, and intensity. We have used our refined foci counting techniques to explore the frequency of recombinant fluorescent cells among tissues and also to compare the difference in frequency between heterozygous animals that have one copy of the reporter (R/+), and homozygous mice that have two copies of the reporter (R/R). For tissues, we observed a high frequency of HR in the pancreas, and much lower levels in the liver and colon, which is consistent with previous studies. Furthermore, consistent with the literature showing that most recombination occurs between sister chromatids rather than homologous sequences, we observed an approximately 2X increase in HR in the homozygous animal for pancreatic and colon tissues. Additionally, we have used this approach to differentiate between large and small foci in the colon, which has provided valuable insights into the rate of HR in transit cells versus somatic stem cells^27^. Taken together, advances described here are immediately useful to researchers studying HR, and it is anticipated that this work will form a foundation upon which it will be possible to analyze additional genetic or molecular changes that can be detected by fluorescence within intact tissue.

## Electronic supplementary material

Supplementary Information

## Data Availability

The source code of the algorithms and the datasets generated and analyzed during the studies included in this paper are publicly available.
