# Supplementary material for: Automated fluorescence intensity and gradient analysis enables detection of rare fluorescent mutant cells deep within the tissue of RaDR mice
Source: Sci Rep. 2018 Aug 14;8:12108. doi: 10.1038/s41598-018-30557-9 (PMC6092416; doi:10.1038/s41598-018-30557-9)
Supplement: Supplementary file 1 — Supplementary Information [file 41598_2018_30557_MOESM1_ESM.pdf]

Supporting Materials: Automated fluorescence intensity and gradient analysis enables detection of rare fluorescent mutant cells deep within the tissue of RaDR mice

Dushan N. Wadduwage[1,2,3], Jennifer Kay[1], Vijay Raj Singh[2,4], Orsolya Kiraly[1,2], Michelle R. Sukup-Jackson[1], Jagath Rajapakse[2,5*], Bevin P. Engelward[1,2*], Peter T. C. So[1,2,4*]

[1] Department of Biological Engineering, Massachusetts Institute of Technology, Cambridge, MA 02139
[2] Singapore MIT Alliance for Research and Technology (SMART) Centre, Singapore
[3] Center for BioImaging Sciences, Department of Biological Sciences, National University of Singapore, Science Drive 4, Singapore 117543
[4] Department of Mechanical Engineering, Massachusetts Institute of Technology, Cambridge, MA 02139
[5] School of Computer Science and Engineering, Nanyang Technological University, Singapore

Supporting section S1: The heuristic algorithm for near-surface foci segmentation

We use a heuristic algorithm to adaptively set the *h-value* that result a realistic foci segmentation in the image intensity pipeline. The resulting foci count from this algorithm is later used to set h-value of candidate foci detection in both image intensity and image gradient branches.

The parameter, *h-value*, often dictates the resulting segmentation from the extended maxima transform (Figure S4A). Small *h-values* causes the faulty recognition of local maxima due to noise and higher *h-values* result in failure to detect some candidate foci. Thus, we take three image statistics to represent the above-mentioned phenomenon, namely, the average intensity value of the segmented foci pixels (higher the value better representation of foci), the total area of the segmented foci (lesser the area better representation of foci without region merging or noise detection) and the number of foci (parameters, normalized to the range [0 1], plotted against a range of *h-values* are shown in the Figure S4B). The goal is to take a fuzzy decision to determine an h-value, which will segment highest number of foci with relatively a high average intensity and a low total area. In order to model the above requirements, we define a scoring function shown below.

$$H\_score_f(h) = \frac{Average\ Foci\ Intensity \times number\ of\ Foci}{Total\ Foci\ Area} \quad \textbf{(Eq. S1)}$$

A representative plot for the above scoring function is shown in the Figure S4C. There is an initial sharp decrease in the number of foci with increasing h-value and then the rate of decrease levels off. Therefore, the scoring function plummets initially and starts to increase. Hence we select the h-value that gives the highest local maximum of *H_score* and perform extended maxima transform on the intensity image.

Supporting section S2: Support Vector Machines

Consider a classification problem of finding a classifier that separates the training set,

$$\{(\boldsymbol{X_i}, Y_i) \mid \boldsymbol{X_i} \in \mathcal{R}^d, Y_i \in \{-1, +1\}, \qquad i = 1,2, \dots, N\}$$

where X_i are the d dimensional input vectors and Y_i are the corresponding labels of the (two) classes. SVMs are a class of decision functions, that separates this training data (and similar new data) in to the two classes. Theoretically, support vector machines, involves projecting the input data in to a higher dimensional space, using a mapping, $\phi(.)$, and finding the optimum hyper-plane in the higher dimensional space,

$$\boldsymbol{w} \cdot \boldsymbol{\phi}(\boldsymbol{X}) + b = 0 \quad \textbf{(Eq. S2)}$$

that separates the data in to the two classes. Then the decision function is essentially,

$$decisionFunction(\boldsymbol{X}) = sgn(\boldsymbol{w} \cdot \boldsymbol{\phi}(\boldsymbol{X}) + b) \quad \textbf{(Eq. S3)}$$

This involves solving the quadratic optimization problem,

$$\min_{w,b,\xi} \left(\frac{1}{2}\boldsymbol{w} \cdot \boldsymbol{w} + C \sum_{i=1}^{N} \xi_i\right) \quad \textbf{(Eq. S4)}$$

subjected to the constraints, $\forall i$

$$Y_i(\boldsymbol{w} \cdot \phi(\boldsymbol{X_i}) + b) \geq 1 - \xi_i \quad \textbf{(Eq. S5)}$$
$$\xi_i \geq 0$$

Here ξ_is are the slack variables introduced to tolerate the misclassification and $C \geq 0$ is penalty parameter on the training error chosen by the user. Solving the dual problem of the above optimization (which is computationally easier than solving its primal problem) gives,

$$\boldsymbol{w} = \sum_{i=1}^{N} \alpha_i Y_i \phi(\boldsymbol{X_i}) \quad \textbf{(Eq. S6)}$$

where α_is are the Lagrange multipliers. Non-zero α_is corresponding to the training data that determines the decision hyperplane and are called the support vectors. Karush-Kuhn-Tucker conditions for the primal problem can be used to determine the value for b [S1]. Thus the final decision function is given by,

$$f(\boldsymbol{X}) = sgn\left(\sum \alpha_i^* Y_i^* \, \phi\left(\boldsymbol{X_i^{SV}}\right) \cdot \phi(\boldsymbol{Y}) + b^*\right) \quad \textbf{(Eq. S7)}$$

here $\boldsymbol{X_i^{SV}}$ are the support vectors and α_i^* and y_i^* are their corresponding Lagrange multipliers and labels respectively. b^* is the values for b found by the optimization.

In the dual problem and the decision function the data appear only as dot products of each other and hence we do not need to define the mapping $\phi(.)$ [S1]. All we need to define is a kernel function in the form of,

$$K\left(\boldsymbol{X_i}, \boldsymbol{X_j}\right) = \phi(\boldsymbol{X_i}) \cdot \phi(\boldsymbol{X_j}) \quad \textbf{(Eq. S8)}$$

This give the user the flexibility of using infinite dimensional mappings such as the one we use, i.e. radial basis function (RBF) kernel.

$$K\left(\boldsymbol{X_i}, \boldsymbol{X_j}\right) = \exp\left(-\gamma \left\|\boldsymbol{X_i} - \boldsymbol{X_j}\right\|^2\right) \quad \textbf{(Eq. S9)}$$

Extensive reference for SVM can be found in for the interested readers.

Supporting section S3: Microscope simulation

Fluorescence imaging was performed using a simulated microscope with a low numerical aperture objective (NA = 0.13) at the emission wavelength, 530nm, similar to the experimental set-up. All simulations were performed using the MATLAB (MATHWorks, Natick, MA) and simulation process is mathematically described below.

Consider $\Omega(x, y, z)$ to be the fluorophores distribution of the locus at the 3D position (x, y, z). The fluorescence excitation was performed using the laser beam with wavelength λ_{EX} and wave number $k_{EX} = 2\pi/\lambda_{EX}$. The 3D distribution of the electric field E, corresponding to projected illumination at the sample space was calculated using the Fresnel's formula as,

$$E(x,y,z) = E_0 \frac{e^{ik_{EX}z}}{i\lambda_{EX}z} \iint \exp\left[\frac{ik_{EX}}{2z}\left\{(x-\xi)^2 + (y-\eta)^2\right\}\right] d\xi d\eta \equiv E_0 \bar{E}(x,y,z) \quad \textbf{(Eq. S10)}$$

Here E_0 is the amplitude of electric field of the projected excitation, z is the axial distance and (ξ, η) is the focal plane of the locus. The 3D intensity distribution can be written as follows:

$$U(x,y,z) = |E(x,y,z)|^2 = |E_0|^2 |\bar{E}(x,y,z)|^2 \equiv I_0 \bar{I}_U(x,y,z) \quad \textbf{(Eq. S11)}$$

Here I_0 is the intensity of the illumination at the specimen. The fluorescence emission intensities for specimen can be calculated as,

$$\Omega'(x,y,z) = \Omega(x,y,z) \times U(x,y,z) \times S(x,y,z) \quad \textbf{(Eq. S12)}$$

Here $S(x, y, z)$ is the scattering function. Assume that fluorescence emission has wavelength λ_{EM}. The 3D point spread function (PSF), $PSF^{3D}(x, y, z)$, is simulated for the detection microscope objective (MO). The intensity function of the detected fluorescence photons at the imaging plane, (x', y'), can be written as,

$$f'(x', y') = \left[\Omega(x,y,z) \otimes |PSF^{3D}(x,y,z)|^2\right]|_{z=0} \quad \textbf{(Eq. S13)}$$

This image is recorded by the CCD camera, where each pixel accumulates electrons in proportion to the number of incident photons and reads out the count. This is a Poisson process and hence Poisson noise was added to the recorded image.

Supporting section S4: Monte Carlo simulations to generate scattering point spread function (sPSF)

a. <u>Monte Carlo simulation process</u>

The light transport process inside a scattering medium, such as tissue, is mainly dominated by two phenomena, light absorption and scattering (see Figure S7A). The absorption coefficient, μ_a, of a scattering medium is defined in such a way that the following expression holds for any path that a photon may follow inside the medium.

$$P(photon\ survives) = e^{-\mu_a L} \quad \textbf{(Eq. S14)}$$

Here, $P(photon\ survives)$ is the probability of the photon's survival (without absorption) and L is the total length of the path the photon travelled inside the medium. A scattering event can happen at any random time during photon's propagation through the medium. The length the photon travels without a scattering event, s, is given by the following expression.

$$s = -\ln(rnd)/\mu_s \quad \textbf{(Eq. S15)}$$

Here, rnd (where, $0 < rnd \leq 1$) is a random number and μ_s is the scattering coefficient of the medium. A scattering event changes the photon's direction of travel by an angle, θ. The probability distribution function of θ is given by,

$$P(\theta) = \frac{1}{4\pi} \times \frac{1-g}{(1+g^2+2g\cos(\theta))^{3/2}} \quad \textbf{(Eq. S16)}$$

Here, g is the anisotropy of the medium. Thus, the random walk of a photon (Figure S7B) through a scattering medium can be probabilistically simulated according to the above equations when the medium parameters, μ_a, μ_s and g are known. In this work we used the Mote Carlo(MC) simulation implemented in the reference S2.

Briefly, the escaping flux density of fluorescence at a point ($\boldsymbol{r}$) on the tissue surface can be described by the following equation [S2].

$$J_f(\boldsymbol{r}) = P_0 \int_{volume} T_x(\boldsymbol{r}')\ \varepsilon C(\boldsymbol{r}') Y\ T_f(\boldsymbol{r}, \boldsymbol{r}')\ dV(\boldsymbol{r}') \quad \textbf{(Eq. S17)}$$

Here, $\boldsymbol{r}'$ is a vector that specifies the position of the incremental volume $dV(\boldsymbol{r}')$ within the tissue, $dV(\boldsymbol{r}')$ is the incremental volume at $\boldsymbol{r}'$ of the volume integration, P_0 is the incident power of the excitation beam, $T_x(\boldsymbol{r}')$ is the transport of source power at the excitation wavelength such that $P_0 T_x(\boldsymbol{r}')$ yields the local fluence rate at $\boldsymbol{r}'$, ε is the extinction coefficient of the fluorophore (using base e), $C(\boldsymbol{r}')$ is the concentration of fluorophore at $\boldsymbol{r}'$, Y is the power yield, $T_f(\boldsymbol{r}, \boldsymbol{r}')$ is the transport of fluorescence power from $\boldsymbol{r}'$ to yield the escaping energy density at the tissue surface at position $\boldsymbol{r}$, $J_f(\boldsymbol{r})$ is the flux density of escaping fluorescence at the surface at position $\boldsymbol{r}$. Mote Carlo simulations calculates statistical averages for $T_x(\boldsymbol{r}')$ and $T_f(\boldsymbol{r}, \boldsymbol{r}')$ based on a large number of photons' random walks. $T_x(\boldsymbol{r}')$ and $T_f(\boldsymbol{r}, \boldsymbol{r}')$ in (4) also takes refraction (and hence total internal reflection) at the air tissue boundary in to consideration. Implementation of the MC simulation, sometimes uses speed of light inside the medium. Therefore, in addition to, μ_a, μ_s and g, refractive index of tissue, n, should also be known. Interested readers can refer to Jacques [S2] for a detailed description of the simulation process.

b. Approximation to tissue parameters

The tissue parameters μ_a, μ_s, g and n were selected from the literature to mimic mouse pancreatic tissue as closely as possible. A simplified version of the model suggested in the reference S3, was used to calculate the above parameters. μ_a can be calculated using the expression below.

$$\mu_a = BS\mu_{a.oxy} + B(1-S)\mu_{a.deoxy} + W\mu_{a.water} + F\mu_{a.fat} + M\mu_{a.melanosome} \quad \textbf{(Eq. S18)}$$

Here, $\mu_{a.oxy}$, $\mu_{a.deoxy}$, $\mu_{a.water}$, $\mu_{a.fat}$ and $\mu_{a.melanosome}$ are respectively the scattering coefficients of oxygenated blood, deoxygenated blood, water, fat and melanosome. B, W, F and M are volume fractions of blood, water, fat and melanoma. S is HGb oxygen saturation of mixed arterio-venous vasculature. Approximations for S, B, W, F and M for pancreatic tissue were selected form the table 3 in the reference S3. In cases where values for pancreatic tissue were not available, the averages over similar tissue types were used. $\mu_{a.oxy}$, $\mu_{a.deoxy}$, $\mu_{a.water}$, $\mu_{a.fat}$ and $\mu_{a.melanosome}$ at excitation and emission wavelengths (512nm and 529nm for enhanced yellow fluorescent protein - EYFP) were extracted from the graphs reported in the reference S3. Thus absorption coefficients at excitation and emission wavelengths ($\mu_{a.X}$ and $\mu_{a.E}$) were calculated.

μ_s and g were reported hard to measure [S3]. Hence, the average value of 0.9 was used for g as suggested in the reference S3. Then μ_s was derived using the reduce scattering coefficient $\mu_s{}'$ and g according the definition of $\mu_s{}'$.

$$\mu_s' = \mu_s\,(1-g)\ \textbf{(Eq. S19)}$$

μ_s' at excitation and emission wavelengths were calculated using the following equation.

$$\mu_s' = a\left(\frac{\lambda}{500}\right)^{-b}\textbf{(Eq. S20)}$$

Approximate values of a and b for pancreatic tissue were chosen from the table 2 in the reference S3.

Finally, an approximation for n for pancreatic tissue was selected by taking the average of the refractive indices of water and dry tissue mass reported in the reference S3.

c. <u>Monte Carlo simulation results</u>

The Monte Carlo simulation was run using the tissue parameters described above and figure S8 shows the resulting point spread functions that were used in the main text.

References

[S1] Burges, Christopher JC. "A tutorial on support vector machines for pattern recognition." *Data mining and knowledge discovery* 2.2 (1998): 121-167.

[S2] Jacques, Steven L. "Monte Carlo simulations of fluorescence in turbid media." *Handbook of Biomedical Fluorescence* (2003): 61-107.

[S3] Jacques, Steven L. "Optical properties of biological tissues: a review." *Physics in medicine and biology* 58.11 (2013): R37.

Supporting tables

Table S1. The list of features

Geometrical features	Intensity features (on preprocessed image)	Gradient features (on Focus flow)
Area	Mean intensity	Mean Focus-flow
Eccentricity	Integrated intensity	Integrated Focus-flow
Circularity[1]	Standard deviation of Intensity	Standard deviation of Focus-flow
Perimeter	Intensity range	Focus-flow range
Convex area[2]	Maximum intensity	Maximum Focus-flow
Relative convex area[3]	Mean boundary intensity	Mean boundary Focus-flow

[1] Circularity: the ratio between the perimeter and the area,
[2] Convex area: the area inside the convex hull for the segmentation of each focus and
[3] Relative convex area: the ratio between the convex area and the area of the focus segmentation.

Supporting figures

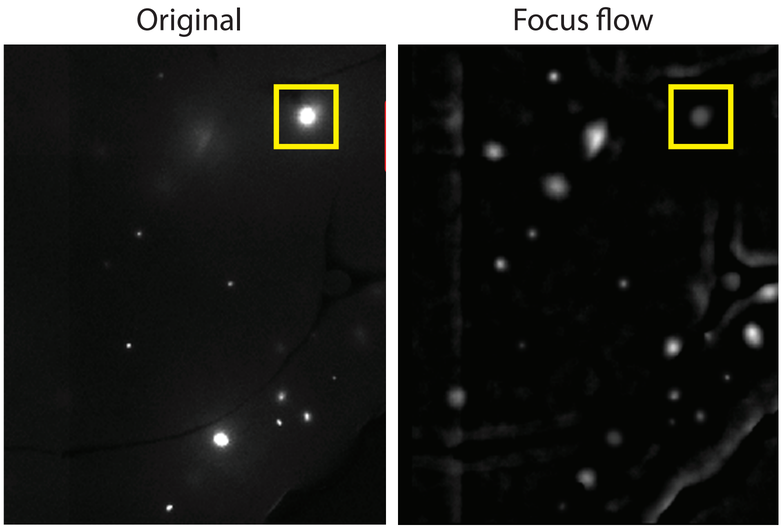

Figure S1. Saturated foci generate weak responses in Focus-flow

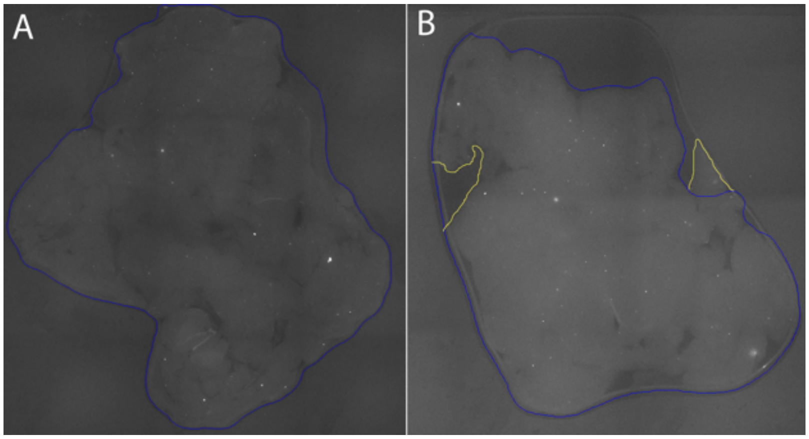

Figure S2. (A) An accurate foreground segmentation – shown in blue - by snakes algorithm. **(B)** An inaccurate foreground segmentation result by snakes algorithm. The segmentation had to be refined using the graphical user interface as shown in yellow.

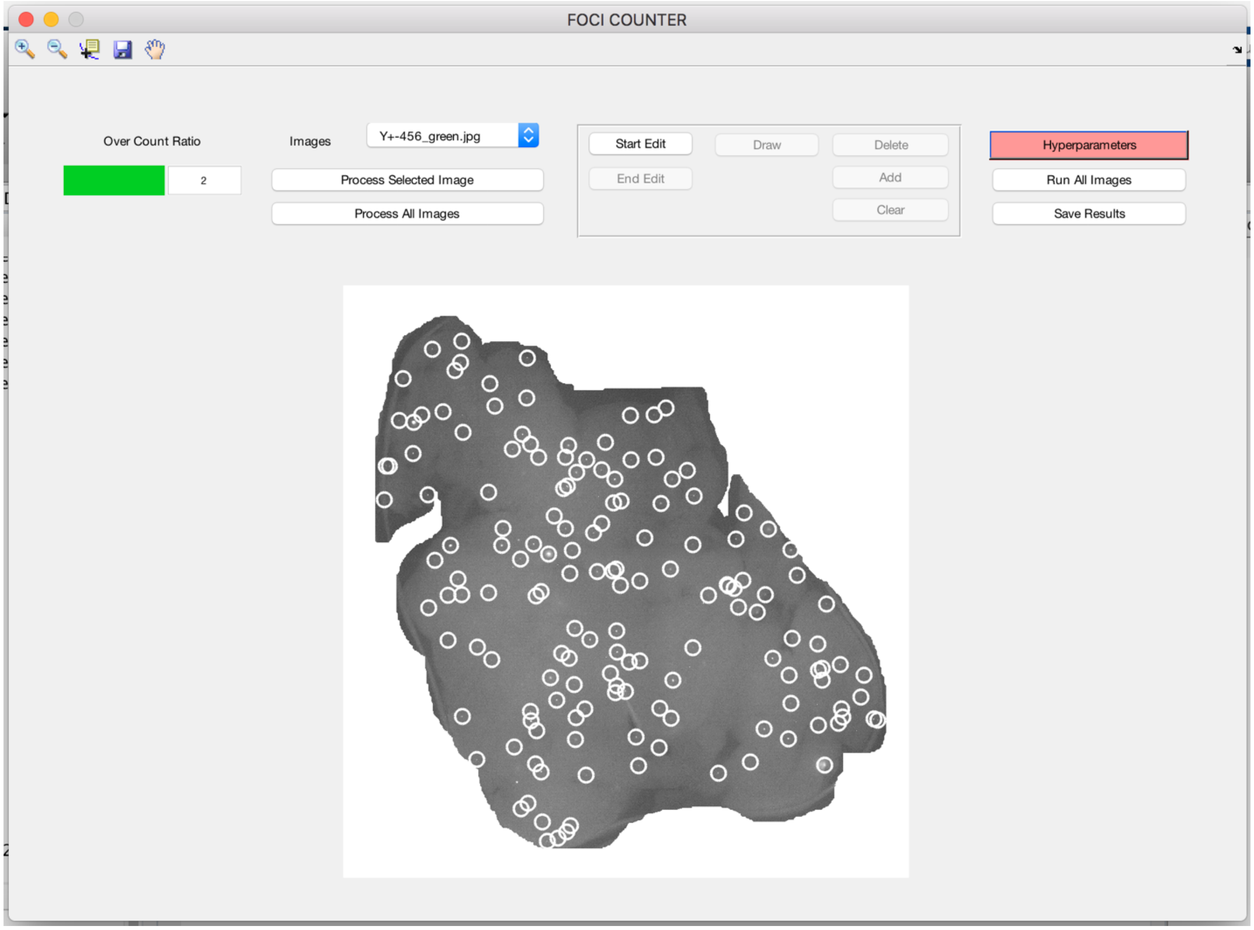

Figure S3. A screenshot of the graphical user interface.

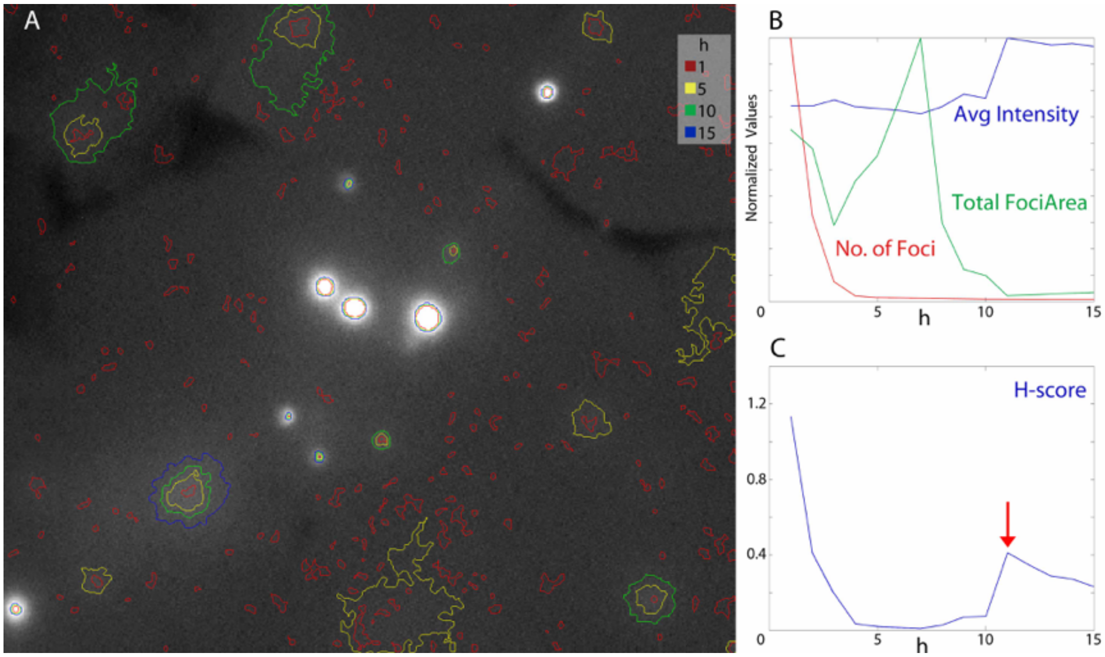

Figure S4. (A) Segmentation resulting from EXMAX in the heuristic algorithm. Different colors show different "*h-values*" (denoted as h=1, 5, 10, and 15) as the parameter. **(B)** Selected image statistics

(normalized to the range [0 1]) plotted against a range of h-threshold values (1 to 15). **(C)** H-score for the three image statistics in 'B' plotted against h-values. The red arrow shows the highest local maximum that corresponds to the selection of h-value.

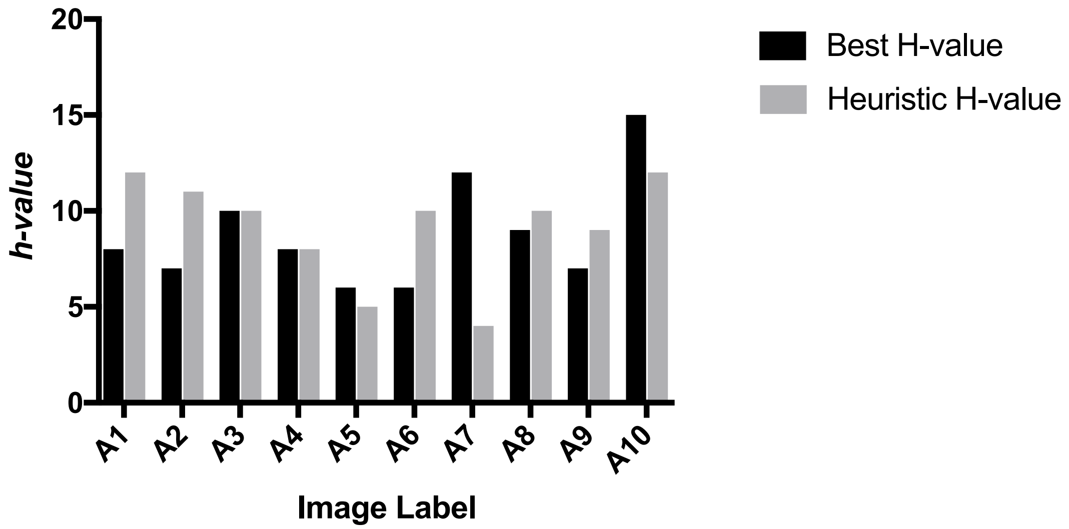

Figure S5. The best *h-values* and the selected *h-values* (by the heuristic algorithm) for a set of 10 images of pancreatic tissue from a single cohort of animals.

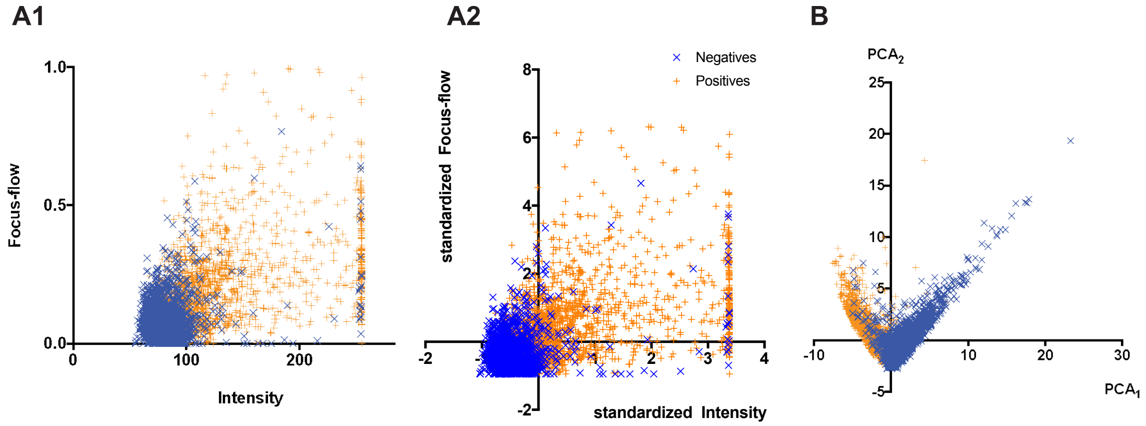

Figure S6. (A) An image intensity and an image gradient feature (mean intensity and mean Focus-flow) plotted for all training instances. Shown in A1 are the original feature values. Shown in A2 are the standardized values of A1. **(B)** The first two principal components plotted for all the features after principal components analysis (PCA).

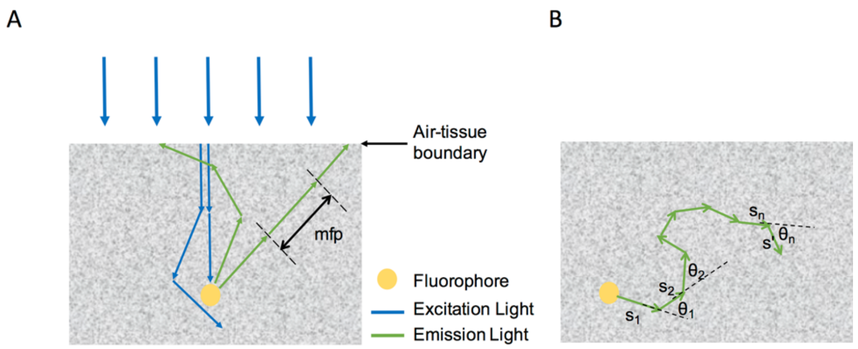

Figure S7. (A) Light transport model inside tissue. Photons of excitation light (shown in blue) enters tissue through the air-tissue boundary and travels towards the fluorophore. Photons interact with tissue either by absorption (quantified using absorption coefficient, μ_a) or scattering (quantified using scattering coefficient, μ_s and anisotropy, g). On average, a scattering event occurs after a mean-free-path and some excitation photons reach the fluorophore and causes isotropic emission photons generation (shown in green). Emission photons, similarly subjected to absorption and scattering, reaches air-tissue boundary. **(B)** A random walk of an emission photon. n scattering events occurs before the photon dies.

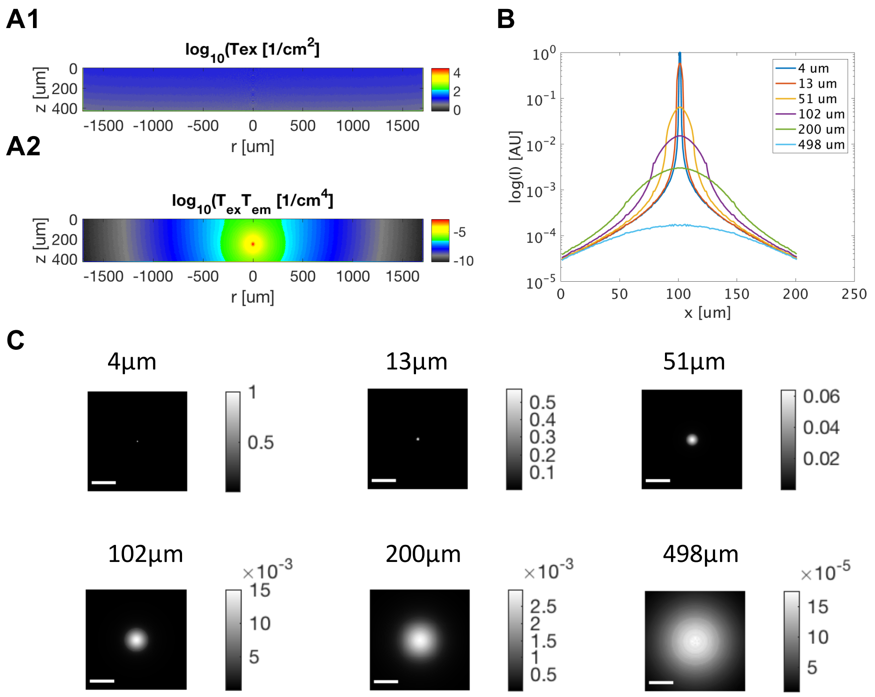

Figure S8. (A1) Monte Carlo simulation of excitation laser light inside a thick tissue specimen. **(A2)** Monte Carlo simulation of emission fluorescence light from a point fluorophore at $250\mu m$ depth. **(B)** Scattering point spread function (sPSF) at different depths. **(C)** 2D representation of the sPSF at the representative depths shown in 'B'
